# A Practical Treatise on Diseases of the Eye

**Published:** 1844-10

**Authors:** 


### ART. XI.

*A Practical Treatise on Diseases of the Eye.* By WILLIAM JEAFFRESON, late Surgeon to the Bombay Eye Infirmary, in the Hon. East India Company's Service, &c.—*London*, 1844. 8vo, pp. 308.

MR. JEAFFRESON tells us, in his preface, that although "he has selected an old subject, he trusts that having treated it somewhat differently, it may not prove altogether uninteresting. If he has painted it," he adds, "in new colours, he hopes these colours may be found true to nature." After this announcement, we felt, we must confess, somewhat disappointed to meet with so very few new views in the course of the work. We are ready to grant, however, that Mr. Jeaffreson does not, in general, write unadvisedly; he has evidently studied, with some attention, the opinions of his predecessors on most of the topics which come before him, and has not hesitated to examine and to judge for himself.

In his introductory chapter, and in several other parts of his work, Mr. Jeaffreson refers, with great complacency, to the extensive opportunities he has had of studying practically the diseases of the eye, especially at Bombay, where, in little more than ten years, he treated, he says, 53,359 cases. Of these, he says, 7334 were cases of cataract; and that out of this number, 5517 were "restored to perfect sight by operation or other treatment." (p. 12.) This is a statement which we do not feel disposed implicitly to receive; especially when he reiterates it at page 16 in the following startling terms. "Out of this number," he says, "nearly seven thousand utterly blind persons were restored to perfect sight; namely, one thousand four hundred and thirty-two suffering from amaurosis, and five thousand five hundred and seventeen affected with cataract." Now, laying out of view the amaurotic cases, it is well known to all medical men, that "an eye affected with cataract," to use the words of Mr. Wardrop, "never becomes perfectly blind;" and it is as certain that an eye, cured of cataract by operation, is never restored to perfect sight. Mr. Jeaffreson may perhaps say, however, that his phrases, "utterly blind" and "restored to perfect sight" are not to be taken quite literally, but are small oriental flourishes, which he had picked up during his Indian residence.

Mr. Jeaffreson belongs to the school of ophthalmologists who are terribly afraid of doing too much, in the treatment of diseases of the eye. He cautions us, therefore, at the outset, against "an eagerness to arrest all morbid processes of this organ, with an activity and impetuosity of treatment," which may sometimes lead the practitioner to sacrifice the patient's constitution to the securing of his sight. "The excessive practice," he says, "to which I allude, is especially that of bloodletting, mercurial treatment, and all those means which tend greatly to lower and impair the general powers of the system." (p. 5.)

Upon this question of active treatment, and especially of active depletion from the system by the lancet, we think the rule a very plain one. In those acute inflammations of the eye, which are attended by a quick full pulse, throbbing pain in and around the eye, such as prevents sleep, and an imminent danger of disorganization, or at least of serious morbid change, of some important part, as in purulent ophthalmia, rheumatic ophthalmia, syphilitic iritis, &c., the lancet should always be employed; while in those inflammations which are not attended by such violent symptoms, as catarrhal ophthalmia, scrofulous ophthalmia, &c., general bloodletting may be abstained from. As for mercury, the rule is, also, in our judgment, a very simple one: When the disease is of that sort which is apt to be attended by fibrinous or purulent effusion into the interior of the eye, make the mouth sore. It is a defective diagnosis, which leads those who are imperfectly skilled in eye-diseases, sometimes to destroy their patients' constitutions, by bleeding and mercury, when they should perhaps prescribe tonics, as in scrofulous ophthalmia; and at other times to leave them with the pupil contracted, adherent, and opaque, from the neglect of venesection and mercury. This last event is very apt to happen in the hands of those who take fright at what they call the patient's "want of power."

Chapter II. treats of the *acute non-specific inflammations of the eye;* and, on the whole, we regard this and the following chapter as the best part of Mr. Jeaffreson's book.

After enumerating the local phenomena of inflammation, "redness, heat, pain, and swelling," Mr. Jeaffreson tells us that "to these signs might have been added increased and painful susceptibility of the nerves of the part affected, or in its neighbourhood, to their customary impressions," (p. 19,) referring, we suppose, to the intolerance of light, which attends some of the ophthalmiæ.

With respect to the pain which attends scrofulous ophthalmia, our author emits an opinion which is new to us, namely, that it is owing to the motion made by the iris in excluding the light. "It is this movement of the iris," says he, "which I believe is the cause of pain under these circumstances, just as to move the finger or toe, in the case of common whitlow or gout, occasions great pain." (p. 21.) Whether this notion has any truth in it, we shall not venture to decide. Certain it is, that dilatation of the pupil by belladonna is generally attended by striking relief to all the symptoms of the disease in question, a fact, however, which does not seem to have come under Mr. Jeaffreson's notice.

The following remarks on the general line of treatment to be followed in the inflammatory diseases of the eye are judicious, and may be quoted as a fair specimen of Mr. Jeaffreson's views:

> "In all cases of ophthalmia the medical attendant should carefully inquire into the cause of the affection, and the previous condition of his patient's system. The more intimately he finds the local affection to be connected with conditions of general health, the more carefully must he endeavour to set the general health to rights, which will constitute the most important part of his treatment as directed to the eye; for however much it may be in his power, by local applications, to restore the eye to an apparent condition of health, such restoration will indeed be but apparent and temporary." (p. 26.)

Mr. Jeaffreson's treatment of catarrhal ophthalmia we should regard as over-active, and as leaning too much to the use of tartar-emetic:

> "The treatment required for acute conjunctivitis, includes the use of the emetic substances, especially tartar emetic, and active purging in the first instance; a dose of calomel, combined or not, as the case may require, with some antimonial preparation, or aperient, followed by the common black draught of senna and salts. If the inflammation be very severe an emetic may be found desirable. After the action of such remedies, our object will be by rest, abstinence, and determination to the skin, bowels, and kidneys, to keep down the force and frequency of the circulation, and with it the local inflammation." (p. 37.)

Mr. Jeaffreson's remarks on collyria coincide pretty much with those of Mr. Travers:

"As [for] collyria, the simple element water constitutes the great base or menstruum of other collyria, and is often sufficient in itself. I am in the habit of recommending it to be used cold, even in the earlier stages of conjunctivitis, and generally with the best effects; it sometimes, however, happens in these as in some other cases, that cold causes a disagreeable, whilst warm applications induce an agreeable sensation to the patient, and it is always well to have respect to the sensations induced, which usually indicate with great certainty the degree of temperature not only most comfortable but most beneficial to the patient." (pp. 38-9.)

One particular of Mr. Jeaffreson's practice surprises us exceedingly; and that is his employment of the preparations of lead, even in cases where they are almost sure to prove highly injurious.

"More important," says he, "than the collyria, are the various substances used to drop into the eye." (p. 39.) "Some of the substances thus used have a direct tendency to induce contraction of the capillary vessels to which they are applied, as the saturnine lotion." (p. 40.) In purulent ophthalmia, he says, "a little tepid water or goulard lotion should be carefully injected under the edges of the lid four or five times a day." (p. 87.) And in ulcer of the cornea he says, "in these cases the liq. plumbi diacetatis dropped into the eye has the best effect." (p. 115.)

The fact being completely established, that when a solution of a salt of lead is applied to the surface of the eye, it immediately undergoes decomposition, so that an insoluble precipitate of chloride of lead is thrown down, which attaches itself to any excoriated or ulcerated spot of the conjunctiva or cornea, adhering thereto tenaciously, and in the healing of the spot becoming permanently and indelibly imbedded in the cicatrice, we had thought the application of lead to the eye was altogether abandoned. Apply Goulard water to an ulcer of the cornea, as Mr. Jeaffreson advises, and the result is almost sure to be a chalk-white opaque cicatrice. Dr. Jacob's remarks on this subject in the fifth volume of the 'Dublin Hospital Reports,' are worthy of the serious attention of all who meddle with the treatment of eye-diseases.

Mr. Jeaffreson refers to certain applications, which he says "owe their efficacy to their secondary effects, after their stimulating powers have passed off, and to their increasing the secretions of the part, by which the blood-vessels are unloaded and relieved. The vinum opii," he adds, "is the agent most frequently employed for this purpose." (p. 40.) We had always understood opium to possess the opposite effect of that which is here attributed to it, namely, to diminish the secretions of all those organs which come under its influence. It would be strange were the eye an exception to the general rule.

Our author informs us, that "the natives of India are in the habit of using a liquid which they drop into the ear in cases of ophthalmia, and which appears to owe its beneficial agency to a diametrically opposite mode of action to that of counter-irritation." (p. 47.) He believes the "practice to be, in many cases, highly efficacious." It is somewhat remarkable, that after a ten years' residence in India he is able to tell us no more of the composition of the liquid thus employed, than that he suspects "it to consist of narcotic and sedative ingredients."

Chapter III. treats of *chronic and specific ophthalmiæ.*

With respect to acute and chronic inflammations of the eye, Mr. Jeaffreson tells us, that "the state of the tears will sometimes mark a distinction, and it is always of importance," he adds, "to inquire whether they feel hot or cold when falling down the cheek." (p. 50.) We have never happened before to hear of cold tears.

The ophthalmia commonly called *pustular*, Mr. Jeaffreson designates as "vesicular or vesiculo-pustular." The most correct name we believe to be that used by Mr. Morgan, viz. *aphthous inflammation* of the conjunctiva.

At p. 59 Mr. Jeaffreson relates, at great length, a common case of scrofulous ophthalmia, which yielded readily to leeches and a solution of nitrate of silver. We notice this case to point out the inconsistency of the treatment, with the clamorous attack on the practice of leeching, in the very disease in question, only a few pages before.

The notice Mr. Jeaffreson takes of intermittent ophthalmia is very short. It is well known that this disease is extremely rare. We should have been glad, therefore, as Mr. Jeaffreson hints that he has had some experience of it, had he favoured us with some account of its symptoms and course.

"Syphilitic iritis," says our author, "as regards the local phenomena to which it gives rise, does not materially differ from the ordinary inflammation of this delicate structure." (p. 67.) This is an assertion to which we cannot subscribe. The tubercles or small abscesses which rise on the surface of the iris, when this membrane is affected with syphilis, differ most materially from any of the symptoms, which attend the idiopathic or any other variety of iritis. In the treatment of syphilitic iritis, Mr. Jeaffreson trusts chiefly to mercury, and expresses a great fear of taking away blood. "The practitioner should be careful," he says, "how he lowers the powers of life in a person labouring under syphilitic disease." (p. 68.) That cases of syphilitic iritis do occur in debilitated subjects, unable to bear either bleeding or mercury, without danger of sinking, we readily admit; but, on the whole, we believe the error commonly fallen into to be quite on the other side. Depletion is too much neglected, and the cure impeded by too stimulating a diet. Most assuredly, the irregular, adherent, semi-opaque pupil, so often left as a sequela of syphilitic iritis, is the result of trusting too much to mercury and too little to the lancet. "I have seen the disease," says a very competent authority, "running on with rapid strides to dangerous hypopion, notwithstanding the full action of mercury, and its further progress at once arrested by a full bleeding from the arm and a blister on the hind-head."*

Under the head of "gonorrheal ophthalmia," Mr. Jeaffreson makes a remark, which requires some modification to be perfectly correct. "In favour," he says, "of the occasional constitutional origin of the disease, it must be borne in mind, that it [gonorrheal ophthalmia] not unfrequently forms part of a chain of secondary symptoms, chiefly of a rheu-

* Monteath's Essay on Iritis, Glasgow Med. Journal, vol. ii, p. 59; Glasgow, 1829.

matic character, which are apt to supervene upon gonorrhea." (p. 71.) Now, the disease of the eye, which originates through the medium of the constitution, is not at all an affection of the conjunctiva, like the ophthalmia which is produced by inoculating the eye with the discharge from the urethra. It is, on the contrary, an iritis of very severe character, attended with a more profuse effusion of lymph than any other variety of iritis; but, under the influence of depletion and mercurialization, susceptible of a cure much more complete than what generally follows syphilitic iritis. We have no evidence of a purulent ophthalmia or a conjunctivitis forming any part of the chain of secondary symptoms, which undoubtedly supervene, in certain cases, upon gonorrhea.

In the active stage of the purulent ophthalmia arising from inoculation of the conjunctiva with gonorrheal matter, Mr. Jeaffreson tells us that "a mercurial course is worse than useless, as tending to break down those constitutional powers of resistance to the disease of which we stand most in need," (p. 73;) and a little farther on, when speaking of the purulent ophthalmia of adults, he says, "In purulent ophthalmia, the mercurial treatment carried to salivation is decidedly objectionable, as increasing the tendency to sloughing and ulceration." (p. 92.) We believe this to be a mere prejudice, and that the treatment so successfully employed by Mr. Muir of Paisley, in the Egyptian ophthalmia, would prove equally efficacious in the gonorrheal or in any other purulent inflammation of the conjunctiva. "I have employed salivation," says Mr. Muir, "in many other cases of ophthalmy with the same happy effects; the pain and inflammation always receding, as the salivary glands became affected, and the sight becoming as strong as formerly. The temporary inconvenience from the mercurial disorder is compensated, not only by the cure of this tedious and dangerous disease, but in many by a better state of health than they for some time enjoyed."* Were we to stop to inquire how it is that mercury proves so beneficial in purulent ophthalmia, we should find the explanation partly in a fact of which Mr. Jeaffreson is perfectly aware. "For it should be stated," he says, "that although the majority of inflammatory affections of the eye are happily slow to extend themselves to other structures than those primarily affected, it is the characteristic of such forms of ophthalmia as induce suppuration to extend themselves rapidly from the outer to the inner and deeper-seated structures of the organ." (p. 84.) Now, in inflammation of the internal textures of the eye, bleeding from the arm and salivation are well known to be our chief resources.

Mr. Jeaffreson is of opinion that in the treatment of eye-diseases, "in proportion to the amount of purulent secretion should we be most wary, most careful, in the adoption of powerful antiphlogistic measures." (p. 76.) Careful we grant, but against the timorous prudence recommended by Mr. Jeaffreson we must enter our *caveat*. His comparisons, drawn from internal abscesses, inflamed liver, purulent secretions from the bronchial mucous membrane, and sloughing compound fractures, are, in our opinion, very little to the purpose. In the acute purulent ophthalmiæ, such as the Egyptian, the gonorrheal, or even the ophthalmia neonatorum, were the oculist to stop, whenever he saw a copious puru-

* Edinburgh Medical and Surgical Journal, vol. vii, p. 245; Edinburgh, 1811.

lent discharge from the conjunctiva, and say to himself, Now, I must use no more antiphlogistic means, what would be the result? The probability is, that in nine cases out of ten, the cornea would become infiltrated with pus, ulcerate, and burst, and the disease end in blindness.

With respect to the purulent ophthalmia of infants, Mr. Jeaffreson observes, that "a disposition exists in the mind of many practitioners to attribute this disease invariably to the inoculation of the eye with gonorrheal poison. Certain it is," he adds, "that a vast majority of such cases occur in that class of society in which such a cause is most likely to be in operation; but at the same time it must be difficult to prove that no such cases ever occur without the operation of this cause." (p. 78.) This last remark is obscurely expressed; but so far as we can make out the meaning of it, we should say there was no difficulty in the matter. Cases of purulent ophthalmia in infants are not at all uncommon, where the mother is perfectly free from gonorrhea.

In the treatment of the disease, our author uses a solution of nitrate of silver, beginning with half a grain to the ounce of water. This might do very well as a wash, with which to remove the purulent discharge, perhaps every two hours; but is far too weak as a drop, to be applied by means of a camel's hair brush, once or twice a day.

In regard to the effects of purulent ophthalmia, as displayed amongst the modern Egyptians, Mr. Jeaffreson, from personal observation, confirms the accounts of Mr. Lane and other travellers. The following account is interesting:

"The number of persons one meets in Egypt who are totally and irrecoverably blind is indeed most frightful; but from my own observations I am disposed to think that more eyes are lost from those chronic forms of disease engrafted upon primary acute purulent ophthalmia, than during the active state of this complaint itself.

"Nothing is more common than to witness in the bazaars and other frequented places in Egypt, the most disgusting and revolting sight of persons going about with great streaks of puriform secretion running down their cheeks, and matted to their beards with dust and dirt, the accumulation of many days, unwashed. Naturally indolent, in common with the inhabitants of most warm countries, they neglect the earlier stages of disease; and amongst the male part of the population at least, the incentive to obtain relief is abated by their desire to escape military service, at the expense of serious mischief to, or even total loss of an eye; and indeed I understood that it was not uncommon for men voluntarily to inflict blindness of one eye, or other serious mischief upon themselves, with the view of escaping their liability to serve in the army. Another apology for the dirt and especial filthiness of the Egyptians may be found in the general scarcity and want of water.

In the hands of an intelligent practitioner, calomel, black draught, and a plentiful supply of water, would alone materially diminish the frequency and virulence of this painful disorder.

The habit of the Egyptians is to crowd themselves much together; their mode of living is very poor; they spend a considerable portion of time in the bazaars and other public places, where the profusion of flies, at all times sufficiently great, is aggravated by the exposure of dates, raisins, figs, and other sweets, by which they are attracted; it is to these flies, carrying the purulent secretion from one eye to the other, that I believe the extension of the disease is frequently due; and I would strongly urge all persons in their travels through Egypt, to be provided with veils for the purpose of keeping off these insects; gauze, or dark-coloured spectacles, may protect the eyes against the glare of the sun, or partially even against dust, but they do not prevent flies from settling on the lids." (pp. 97-9.)

We must confess we are surprised at Mr. Jeaffreson's notions respecting variolous ophthalmia. We had believed that it was established beyond the shadow of a doubt, that the eruption of smallpox never appears on the conjunctiva; that the cornea is never the seat of a smallpox pustule; that the destruction of the eye, which is apt to follow smallpox, is never a primary but always a secondary affection; that it is after the swelling of the eyelids has fallen, the scabs have dropped off, and the eyes are open, that variolous ophthalmia arises; and that the affection of the cornea, so apt to lead to loss of sight, is an abscess in its substance, not a pustule on its surface. The reverse of all this is set down by Mr. Jeaffreson, (p. 101,) by whom everything that Gregory, Guersent, and Marson have said on the subject is either totally unknown or contemptuously neglected.

A very striking defect in Mr. Jeaffreson's work is, that no separate notice is taken of corneitis. Choroiditis is also omitted.

The fourth chapter is devoted to *structural changes the result of inflammation,* and to *certain affections of the humours.*

"Ulcer of the cornea," says our author, "may keep up inflammation of the surrounding parts, just in the same way as an ulcer of the leg, in which case the treatment best suited for the ulcer is best adapted for the inflammatory action of the surrounding parts. (p. 105.) The fact is true, but the illustration is poor and lamely expressed, serving only to recall to mind the felicitous comparison drawn by Andral,* between the affections of the eye, in which inflammation is by turns cause and effect, and similar phenomena, connected with tubercles in the lungs.

Mr. Jeaffreson boasts that it was his "good fortune to be educated under the justly celebrated and talented surgeon Mr. Abernethy." (p. 7.) It may have been from this source, that Mr. Jeaffreson picked up the notion held by John Hunter, that ulceration is a process instituted in disease to prevent the occurrence of a more serious evil, namely, mortification. This opinion Mr. Jeaffreson has outrageously caricatured, when he tells us that his "own conviction is that ulceration is properly, so to speak, a restorative process," (p. 112;) a remark which puts us in mind of the maxim of a talented old teacher of our own, that whenever a man qualified his statements with "so to speak," or "as it were," we might be pretty sure he was talking nonsense. "Why not, then, it may be said," exclaims Mr. Jeaffreson, "allow nature her free course in the processes of ulceration and sloughing? And so we do," adds he. Now we do no such a thing. When called to a case of ulcer of the cornea, knowing well, to use the words of perhaps the man of greatest talents that ever wrote on eye-diseases, that ulcers of this part, "abandoned to themselves or improperly treated, spread rapidly, become deep, and destroy the parts which they occupy," * we immediately set to work to limit the inflammation on which the ulcer depends, and promote the healing processes of granulation and cicatrization, by every local and general remedy in our power. If ulceration is "a restorative process," as Mr. Jeaffreson calls it, we should like to know what granulation and cicatrization will be in his vocabulary.

* Clinique Médicale, tome ii, pp. 185-6; Paris, 1829.

† Scarpa's Treatise on the principal Diseases of the Eyes, translated by Briggs, p. 214; London, 1818.

Abandoning his principle, that ulcers are good things, Mr. Jeaffreson in a couple of pages necessarily comes to recommend practical views, perfectly consonant to the generally received opinion :

"The most active steps should now be taken to restore the parts primarily affected to a more healthy condition, which may be affected by the application of the caustic, or its solution, to the ulcer—or when the ulceration is accompanied by a granular state of the lids, as is not unfrequently the case, by the use of blue-stone. Some local stimulants may be generally used with advantage, as dropping the vinum opii into the eye; but sometimes the immediate stimulating effect of the blue-stone or caustic will be sufficient to impart fresh vigour to the vessels of the ulcer, whilst they act as powerful sedatives to the other parts of the eye." (p. 115.)

The last notion would come nearer the truth, were it reversed, thus : Sometimes the effect of the blue-stone or caustic will act as a powerful sedative to the ulcer, while it imparts fresh vigour to the vessels of the other parts of the eye. The caustic, as Scarpa pointed out, destroys the naked extremities of the nerves in the ulcerated part, and quickly takes off the morbid excess of sensibility. This is something like sense, whereas to suppose that caustic will at once invigorate the vessels of the ulcer, and act as a sedative to the other parts of the eye, is as if one were to try blowing hot and cold at the same time.

Speaking of common staphyloma, Mr. Jeaffreson tells us, that "in this form of disease the iris frequently becomes adherent to the posterior surface of the cornea," (p. 123 ;) but the fact is that such an adhesion, partial or total, according as the staphyloma is partial or total, is a constant part of the disease.

Staphyloma pellucidum, a term applied by some authors to conical cornea, is used by Mr. Jeaffreson to signify hydrophthalmia, which must only give rise to still further confusion.

With respect to artificial pupil, our author informs us, that

"The most suitable operation for all cases consists in the division of the cornea by the knife, as in the operation for extraction to a sufficient extent as to allow of the introduction of scissors adapted for the purpose, by which a portion of the iris is to be cut away, which will generally, as a matter of necessity, be of a somewhat triangular shape, the segment of a circle; a minute hook will also be necessary, either to hold the iris whilst dividing it, or to remove it after its section." (p. 135.)

Now, how are scissors to be safely introduced, we would ask, so as to cut through the iris, in cases where the crystalline lens is transparent? The thing seems impossible. Mr. Jeaffreson would probably tell us, that in cases where there is no adhesion or other disease of the iris, and where the lens is healthy, the iris will prolapse, so that it will not be necessary to introduce the scissors; but he mentions nothing of this in his book. To attempt to lay hold of the portion of iris, too, by means of a hook, after it is cut by scissors, we hold to be an absurdity. If a portion is to be cut out and removed, it must be laid hold of with the hook before the section is made, not after. Should Mr. Jeaffreson reply, that that is what he meant, then why is it not perspicuously and expressly stated ? Referring to the elaborate discussions on artificial pupil to be found in systematic authors, he observes very innocently that there is "some risk of confusion in these very minute details." We apprehend there is much more risk in such meagre general directions, as he himself has favoured us with on the subject.

Chapter v. embraces the *affections of various appendages of the eye.*

For granular lids, Mr. Jeaffreson recommends blue-stone, but there seems some discrepancy between "the free application" of it, which he says "is generally sufficient," and the direction given in the very next sentence, that it should be "passed once or twice lightly over the parts." The following remarks are judicious, and are fully borne out by general experience:

"This, indeed, may be repeated every other day, and an astringent collyrium be used; but I have repeatedly observed, that unless considerable attention be paid at the same time to the general health, such treatment is either very long in effecting its purpose, or sometimes even utterly useless or injurious. Purgatives, alteratives, tonics, proper attention to air, exercise, and diet, constitute the main objects of general attention." (p. 139.)

We cannot so fully concur in the general treatment recommended in cases of tinea and lippitudo. We agree with our author, that "purgatives will generally be required;" that "a light and nutritious diet" should be given; and that "regular habits and proper exercise in the open air should be enjoined," (p. 142;) but even "the moderate use of such stimulants as table-beer or wine," we consider more likely to aggravate than to abate the affections in question.

The sections on trichiasis and entropium are very imperfect. "I know of no remedy," says Mr. Jeaffreson, "for trichiasis, properly so called." (p. 145.) "No measures with which I am acquainted can, with certainty and confidence, be employed to prevent the further growth of these lashes in their inverted position." He recommends, therefore, that the inverted eyelashes should merely be plucked out from time to time. He just hints (p. 148) that an operation in which the bulbs of the cilia are extirpated, has been had recourse to by some practitioners, but seems to regard it as rarely necessary, whereas it is in trichiasis, that the operations of Vacca and Jäger prove the most satisfactory means of radically removing a very troublesome complaint. He tells us, that for entropium, the milder and simpler operation of clipping out a fold of integuments obviates the extirpation of the bulbs of the cilia. In the acute variety, no doubt, it does; but in the chronic, something more must be done, for the clipping out of a fold of integuments may be repeated over and over again, but still the inverted edge of the lid will brush upon the eyeball, as was long ago shown by Sir Philip Cramptom, in his Essay on the Entropeon. On this subject, Mr. Jeaffreson has evidently much to learn, and we would recommend to his perusal an excellent paper on the causes and cure of the diseases in question, by Mr. Wilde, in the 'Dublin Journal of Medical Science' for March, 1844.

The important subject of ectropium, Mr. Jeaffreson dismisses in two pages. His directions for the treatment of this disease form the most slovenly and useless part of his book.

"Ptosis consists in the dropping of the upper lid, which the patient is unable by any voluntary effort to raise. It depends upon paralysis of the muscles whose office it is to raise the lid; and may therefore be due to important forms of cerebral disease. It is occasionally, however, congenital, and not unfrequently depends upon affections of the nervous or muscular fibre exterior to the brain itself, being one specimen of that form of facial palsy which Sir Charles Bell was the first to discover was not dependent upon any mischief within the cranium." (p. 151.)

We suspect Mr. Jeaffreson is here confounding palsy of the face, which Sir Charles Bell showed frequently to depend on disease of the portio dura, independent of any mischief within the cavity of the cranium, with ptosis, on which Sir Charles says little or nothing in his papers on the nerves. Ptosis, or palsy of the upper eyelid, except when it arises from an injury of the levator palpebræ superioris, or of the branch of the third nerve, which stimulates this muscle, always appears to depend on a cause lodged within the cavity of the cranium, never on one placed without. Exposure to cold, acting on the portio dura, as it is escaping from the Fallopian aqueduct, will produce palsy of the face, but no such cause affecting the third nerve after it leaves the cavity of the cranium, ever, as far as we know, produces ptosis.

On the subject of cutting for strabismus, Mr. Jeaffreson makes the following remarks:

"This operation is an ingenious adaptation in the case of deformity of the eye, of the more generally applicable operation of division of the muscles or tendons. It must, however, be remembered that there is this great difference between the applicability of the operation in this instance and in the various other deformities to which the frame is liable, club-foot especially; that whilst in the latter case, mechanical means can be used to secure the parts in their natural position when thus freed from the restraint which has been removed; and whilst active bodily exercise is afterwards used, under proper directions, and at first with the application of mechanical apparatus for securing the natural position of the parts; in the case of the eye all must be nearly or entirely left to chance." (p. 160.)

Mr. Jeaffreson seems quite unacquainted with the fact, that strabismus always implicates both eyes, and to be cured by operation, requires the two adductors, or the two abductors, to be divided, according as the obliquity is convergent or divergent. We cannot admit the correctness of his statement, that "in the case of the eye all must be nearly or entirely left to chance." True it is, that no mechanical apparatus needs, in general, to be applied after the muscles are divided; but much may be done to secure the natural position and natural action of the parts, by what is termed orthophthalmic practice, a very curious and interesting subject, the true principles of which were first developed by Mr. Elliot of Carlisle. Mr. Jeaffreson remarks, that the "operation has been too frequently performed in compliance with the fashion of the day." Perhaps it may be so; but what is a greater evil, it has in general been so badly performed, that we recognize at the first glance, the unfortunate victims of the squint-clipper, by a leer which gives to their countenance an expression really hideous, and not unfrequently with one of their eyes half dislocated from its socket. This is the result of a threefold error: first, digging too deep, and cutting the muscle too far from its insertion; secondly, operating only on one eye, instead of dividing the parallelization between the two; and thirdly, neglecting or misdirecting the subsequent exercise of the eyes.

Chapter VI introduces us to the *affections of the nerves of vision.*

The following is an interesting case of amaurosis, owing, probably, as Mr. Jeaffreson says, to cerebral congestion, and cured by an attack of cholera:

"Captain F., commanding a troop of cavalry at Hyderabad, in his Highness the Nizam's service, whilst on horseback, was suddenly struck by a coup de soleil. He was conveyed home in a palanquin, and remained some days in a state of almost total insensibility. The most active antiphlogistic treatment was used by his medical attendant; but the sight, which was from the first entirely lost in both eyes, did not return with the gradual subsidence of the other symptoms. Soon after this he was advised to come to Bombya, a distance of three or four hundred miles, to consult me. He arrived in a most debilitated state, and so entirely blind as not to be able to distinguish light from darkness, not being able to discern the situation of a window, although opened and in the strongest light: although the vacant stare, so peculiarly characteristic of amaurosis, was well marked in this case, yet the iris was obedient to the stimulus of light, contracting and dilating, but neither to the same extent nor with the same activity as in health.

"He was put upon a steady mercurial treatment, strong stimulating applications were made to the eyes, and frequently repeated blisters to the temples. No benefit arising from this plan of treatment, it was discontinued for a course of tonics, which proved equally unsuccessful. He returned to Hyderabad, and in consequence of his total blindness was obliged to retire from the service.

"Having always given it as my opinion, that so long as the mobility of the iris continued, the case ought not to be abandoned as entirely hopeless; at the expiration of a year and a half, Captain F., being again anxious to consult me, came a second time to Bombay. He arrived quite an altered man in appearance, having ridden all the way on a rough-paced elephant. The eyes, however, were in the same state as when I first saw him, and no improvement in vision had occurred. I again repeated the former plan of counter-irritation by blisters, local stimulants, as of electricity, and put him upon a course of quinine, with generous diet; and, as he was a man of remarkably abstemious habits, I ordered him to take considerably more than his usual allowance of wine. He was also desired to take hard and regular exercise on horseback, before and after sunset, along the beautiful sands of the Back Bay, and to use cold sea-water shower-baths. During this state of things, I was one morning suddenly called upon to attend him immediately, and found him labouring under severe and well-marked cholera. Under proper remedies he rallied, when, to our mutual satisfaction and surprise, with the subsidence of this formidable malady came a return of sight, which after a few days became as perfect as ever. The cure was clearly due to the cholera itself, and not to any remedies prescribed by me for that disease." (pp. 181-3.)

The following remarks on the danger of carrying the depleting and reducing plan of treatment too far, even in those cases of amaurosis in which there is reason to suppose it to arise from pressure or from inflammation, appear to be very judicious:

"We now come to speak of an entirely different class of circumstances, as the cause of amaurosis, and that too of no unfrequent occurrence; I allude to debility, exhaustion, and draining of the blood, whether by its direct abstraction, by means of mechanical injury, the lancet, or other causes; or to the slower but equally sure process of drainage, arising from deficient supplies, or exhausting discharges or secretions. The exact state of the retina, optic nerves, and brain, under these circumstances, it is difficult to explain, or why the powers of vision should, of all others, in this particular form of disease be so singularly and prominently affected. That these nervous structures should suffer proportionately with all others in the deficient supply of their vital fluid, and should therefore be proportionately enfeebled in their functions, can be readily understood; but matters go further, vision is not simply impaired, but frequently entirely lost, whilst the susceptibility to their ordinary impressions of other parts of the nervous system may remain unimpaired. Bearing in mind this particular form of amaurosis, I have always been especially careful in adopting that ultra-antiphlogistic treatment for other forms of the affection, which has been by some so unhesitating recommended, and so unflinchingly practised. I have felt disposed to ask myself the question, how far (setting aside the particular condition to which the disease may be due) the continued perseverance in a powerful course of antiphlogistic treatment may render the retina, optic nerves, and brain, incapable of benefiting, by the removal of such condition, even supposing its removal to be within the power of such treatment? Let me be understood: I mean to say, that supposing gutta serena to be due to the pressure of a tumour, to inflammation, to cerebral congestion, to effusion of blood, serum or lymph; and supposing, again, that bleeding, purging, starving, calomel, antimony, &c., had the inevitable power of removing such causes, what benefit would acrue if, in removing them, the nerves of vision become themselves enfeebled and paralysed, incapable of receiving and transmitting their appropriate impressions? If such be even but occasionally the result of this plan of treatment, and considerable experience has convinced me that it is, some useful cautions may be learnt respecting the limits to which such treatment may, with propriety, be carried, even in cases which seem most adapted for it." (pp. 183-5.)

Chapter VII. is devoted to *cataract*, which subject is introduced to our notice by some remarks on the natural structure of the parts concerned.

"The crystalline lens has an outer investing membrane, known by the name of the capsule of the lens, a membrane perfectly transparent in the healthy condition of the part; a limpid and clear fluid, within this membrane, separates it from the lens itself." (pp. 204.)

Now, there is no such fluid in the healthy living eye. On the contrary, the capsule adheres pretty firmly to the surface of the lens. "I do not believe," says Dr. Jacob, "that any such fluid exists in a natural state." "In the eyes of sheep and oxen, when examined a few hours after death, not a trace of any such fluid can be detected."* Dr. Werneck of Saltzburg discovered on the inner surface of the capsule a distinct layer of nucleated corpuscles, which serve as the medium of union between the lens and the capsule.†

Speaking of the examinations necessary for forming a diagnosis of cataract from glaucoma and amaurosis, Mr. Jeaffreson says, "such examinations will be much facilitated, and rendered more decided, by the artificial dilatation of the pupil with belladonna, and the use of a powerful magnifying-glass." (p. 208.)

If the magnifying-glass is looked through, it will prove of little or no use; if employed to throw the light into the pupil, it will be serviceable.

Not a syllable is said by Mr. Jeaffreson of Purkinje's beautiful adaptation of the catoptrical properties of the crystalline, to the determination of its transparency or opacity, although it affords an easy and infallible test, by which to decide on the existence of cataract or of amaurosis.

We believe there never has occurred any well-authenticated instance of the diminution, much less the removal of a lenticular opacity, by natural nor by artificial means, while the substance of the lens was preserved. Mr. Jeaffreson is inclined to think, however, that the thing is possible.

"I believe that at one period the too prevalent habit of studying diseases in respect of their local character, rather than their constitutional relations, has led to the adoption of that very class of remedies in the treatment of cataract which were rather calculated to do harm than good; hence the general opinion that the disease is beyond the influence of all remedies is true in respect of those which have been most frequently tried. For the very agents employed have been those most likely to impair the functions of nutrition in the lens and capsule, by still further lowering the general health. Such are drastic aperients, local bloodletting, counter-irritation, mercurial preparations, &c. Whereas the means which are most likely

* Cyclopædia of Anatomy and Physiology, vol. ii, p. 200; London, 1837.
† British and Foreign Medical Review, vol. vi, p. 206; London, 1838.

to arrest the morbid processes on which cataract depends, are simply those which impart tone and vigour to the whole system. The treatment, therefore, which I would advise does not consist in any one particular medicine or local application, but in assiduous attention to the general health of the patient, the use of suitable aperients, alteratives, and tonics; aided by a careful management of the eyes themselves; and I have generally found benefit from copious ablution of the temples, eyes, and cheeks with cold water. This view of the subject, too, may not form an entirely useless hint in the cases of those who have an hereditary tendency to this affection, and who consequently are the more likely to apply for the earliest assistance on the part of the profession." (pp. 218-9.)

Mr. Jeaffreson gives, at page 221, a case as an instance of the spontaneous cure of cataract, in consequence of improvement of the general health; we do not think it conclusive.

Mr. Jeaffreson's account of the operations for cataract is too general. Speaking of extraction through the cornea, he tells us that "oculists differ in opinion as to which half should be divided, some recommending the upper, others the lower. The operator," adds he, "must of course have decided this point before proceeding to the operation," (p. 228;) a proposition from which few will dissent. We cannot admit, however, the following statement: "It must be remembered that the operation upwards on the left eye requires to be performed with the left hand." (p. 229.) On the contrary, whether the patient be in the sitting or in the lying position, the right hand serves perfectly well for opening either the upper or the lower half of the left cornea. In this case, the operator stands before the patient, while the assistant, placed behind the patient, raises the upper eyelid. If it is the right eye which is to be operated on, the assistant may stand before the patient, and depress the lower eyelid, while the surgeon, taking his place behind the patient, raises the upper eyelid with his left hand, and with the right opens the upper or the lower half of the cornea. Such, we think, is the mode generally followed by those who are not ambidexter.

The directions given by Mr. Jeaffreson for the performance of depression, are neither sufficiently definite, nor perfectly correct. He says, the needle is to be introduced through the sclerotic, "within a line or two of the junction of this membrane with the cornea, and slightly above the transverse line." (p. 231.) Now, it is not a matter of indifference, as Mr. Jeaffreson seems to think, at what precise distance the needle is introduced. Mr. Abernethy, in his lectures, used to direct the needle to be plunged through the sclerotica, as far as one could from the cornea, because, he said, the degree of inflammation was always in proportion to what was done to the front of the choroid, the ciliary processes, or the iris. But he did not advert to the danger of wounding the retina, if the puncture was at the distance of three tenths of an inch from the edge of the cornea, which is very much within the limit of "as far as you can from cornea."* If the puncture is at two tenths of an inch from the cornea, we should conceive the distance a very good one, as both the retina will be avoided, and the folded part of the ciliary body. But if the puncture is made at one line's distance from the cornea, both the gangliform ring of the choroid and the ciliary processes will be wounded. Wounding the ciliary processes we should not consider of much importance, for they seem mere accumulations of blood-vessels, but the gan-

* Abernethy's Lectures on Anatomy, Surgery, and Pathology, p. 518; Lond. 1831.

gliform ring is a part eminently supplied with nerves, and we have known an accidental puncture of it to be followed by tremulous iris and amaurosis. It should, therefore, be avoided.

Mr. Jeaffreson states no reason why the needle should be introduced "slightly above the central transverse line" of the eye. But there is a very good reason why it should not be introduced there, but exactly in the equator; and that is, that the long ciliary artery divides into two branches, at the distance of three tenths of an inch from the cornea, forming a fork, in the mid-space of which the needle should be passed, so as to avoid both the one branch and the other.

Some modification, Mr. Jeaffreson tells us, of the proceeding followed in depression, constitutes "what is technically called reclination. In this case," adds he, "the lens is not pressed directly downwards, but is turned, as it were, upon its axis." (p. 232.) There is surely some misapplication of terms here; for the axis of a lens is the line joining the middle points of its two opposite surfaces; and to turn the crystalline on its axis, while it remained in the eye, would be rather a difficult thing to do, however easy it might be if it were out of the eye, and stuck upon a pin. Mr. Jeaffreson complains of the *minutiæ* which are to be found in "the more lengthy works of many eminent authors," as "rather calculated to puzzle the practitioner than to assist him," (p. 285;) but we cannot say we have any complaint to make of *minutiæ*, if they be correct; whereas any practitioner trying to turn the lens, "as it were, on its axis," would certainly find himself at fault.

Our author's account of the operation of division through the sclerotica is altogether antiquated. No one, now-a-days, uses a chisel-shaped needle, such as that employed by Sir William Adams, or attempts to slice the lens into pieces, as Mr. Jeaffreson directs. (p. 232.) A round-necked needle is the instrument employed by the operators of the present day, with which the anterior capsule is lacerated, so as to allow the aqueous humour to act on the lens, but without displacing the lens from its situation, or interfering with it in any way whatever. This is the method of proceeding in at least the first operation, for the cure by solution generally requires two or more operations. The absorption of the lens, then, goes on for a certain length of time. In about two months the operation is repeated, the capsule is again lacerated—especially if its fragments seem to have coalesced—and the lens, now become friable by the action of the aqueous humour, is also broken up. The fragments of the lens now dissolve more readily; and probably, in two months more, the pupil is clear.

In his preface Mr. Jeaffreson had already prepared us for something new and important on the cure of cataract by operation:

"In the chapter on cataract the author has advocated an operation of the successful application of which he has had ample experience, and to the easier performance and superior success of it, as compared with the operations in general use, he especially wishes to draw the attention of his professional brethren." (Preface, pp. viii.)

At p. 226 we meet with another note of preparation: "In addition to these ordinary modes of operating, there is yet another method to which it is my earnest desire particularly to call the reader's attention. It is a modification of that by couching and solution."

At last the "mixed operation advocated by the author" bursts upon us. Can the reader guess what this "fourth method of operating" consists in, for which "I am hardly, perhaps, entitled," says Mr. Jeaffreson, "to claim for myself the merit of originality, although, as far as I know, this plan has never been advocated by any practical oculist?" (p. 234.) But it is fair to let the author speak for himself:

"The instrument I use is an ordinary couching-needle, with a double cutting edge, as in the operation for solution. The needle being introduced as before described, when speaking of couching and solution, I first endeavour to cut up as much of the lens in situ, without disturbing it from its natural position, as possible. If the cataract be of the softer kind, it of course yields to the needle, and the operation then becomes simply that by solution; if it be so hard that it cannot be cut up, the capsule at least is freely lacerated, and then the point of the needle being raised, is made very gently to press the lens downwards to the extent of a few lines only, and just sufficient to admit of the entrance of a few rays of light. It rarely happens, however, that the cataract is so uniformly hard but that some portion of it at least may be thus cut up. The lens being held in this position for a few seconds, the needle is then carefully withdrawn. Belladonna should now be kept constantly applied to the orbital ridges, for the twofold purpose of freely admitting the access of the aqueous humour to the lacerated lens, and for facilitating the vision of the patient during the process of absorption." (pp. 234-5.)

Mr. Jeaffreson's operation, then, is this: he tries to divide the lens; but if he finds it too hard to be divided, he depresses it a little, so as to allow the patient to see over the top of it, and leaves it in this position to be acted on by the aqueous humour.

We consider Mr. Jeaffreson's operation objectionable in the first step he takes. If the cure of a cataract is to be effected by solution, there is no use whatever in attempting to cut the lens up; all that is requisite is to lacerate the capsule, and allow access to the aqueous humour. The cutting up of the lens, of which Mr. Jeaffreson is so fond, cannot be effected, if the lens is even only of ordinary consistency, without displacing it from the cavity of the capsule; so that it either falls forward into contact with the iris, or is pushed through the posterior capsule, somewhat into the vitreous humour.

After working away at the lens, then, for some time with his double-edged needle, "if it be so hard that it cannot be cut up," he depresses it a little. Now, in this new situation it will either dissolve or it will not dissolve. If incapable of absorption, it will only prove a source of irritation, and will require to be removed at some future time. If it dissolves, it would have done so had it been left in its natural place, with the front of the capsule merely lacerated. So far as the ultimate cure is concerned, we see no possible advantage to be gained by the semi-depression which Mr. Jeaffreson advocates. The only thing that can be said in its favour is, that it allows the patient a little sight from the first; but we should consider this advantage greatly overbalanced by the danger of the dislocated lens exciting inflammation, by its pressing on the iris or on the ciliary processes. If the operator attempts the cure of cataract by solution, we are of opinion that he has good reason to congratulate himself when he finds the exposed lens to keep its seat within the circumference of the capsule; and that, at the first operation, at least, everything should be most carefully avoided which tends to displace it, in any direction or in the smallest degree.

Chapter VIII. is of a miscellaneous nature, including *malignant diseases*, *mechanical injuries*, *artificial aids to vision*, and several other topics.

Fungus hæmatodes of the eye is described by Mr. Jeaffreson as "commencing probably in the expanded retina," (p. 265); whereas it sprouts from the papilla conica of the optic nerve, and has been found filling the whole cavity of the eyeball, while the retina remained entire.

"My own opinion," says Mr. Jeaffreson, "is very confident and decided that, in the case of fungus hæmatodes, occurring in early life, the operation [of extirpation], if performed without delay, may be perfectly successful. Many cases have occurred to confirm me in this opinion, one of which will be presently related; nor is it unsupported by many high authorities." (p. 264.)

It would have been more satisfactory had Mr. Jeaffreson stated whether the "many cases" which confirmed him in this "opinion" had occurred in his own practice, or in that of others. As for the case of a Hindoo child which he relates (p. 271), and whose eye he extirpated, it is to be observed that the tumour was of a "brown and dusky hue," and that "no blood-vessels were visible on its surface." Now, the bright yellowish surface, strewed with blood-vessels, though it does occasionally occur also in non-malignant tumours, is, we believe, an invariable attendant on fungus hæmatodes.

It is also to be regretted that Mr. Jeaffreson has not given us the names of any of the "many high authorities" by whom the opinion is supported, that the operation, performed without delay, may be perfectly successful. We must confess we are unacquainted with them, and feel inclined, after a careful examination of the subject, to coincide with the judgments of Mr. Travers, Mr. Lawrence, and Professor Syme on this very important practical question:

"I have extirpated the eye affected with medullary cancer in several instances; but I am not acquainted with any case in which the patient, who has survived two years, has not been revisited by the disease." (Travers.*)

"Our present experience is very discouraging, and leads to the inference, that the operation, even in an early stage, is unavailing." (Lawrence.†)

"In adding another instance of unsuccessful extirpation of the eye for medullary degeneration to the many which are already upon record, I cannot refrain from expressing my conviction, that it would be better, both for the interests of humanity and the credit of surgery, if this operation were entirely abandoned. ........If it could be proved that excision had been successful in a single well-authenticated case of medullary tumour of the eye, it would be wrong to refuse the chance of benefit from the operation. But if the result of numerous trials, under every variety of circumstances, has been uniformly unfavorable, it must be cruel to repeat the painful experiment any longer." (Syme.‡)

We are puzzled by Mr. Jeaffreson's "common enlarged sebaceous follicle," "frequently observed on either lid," and "readily removed by merely drawing a lancet or cataract-knife across it, and squeezing out its contents." (p. 275.) Is this the fibrinous tumour, or what is called *chalazion*, and which tends to the inner surface of the lid; or is it the albuminous tumour, called *molluscum*, which studs the outer surface; or is it *bonâ fide* encysted tumour, which the investigations of Sir Astley Cooper proved to be an enlargement of a sebaceous follicle? After opening it in the manner described, Mr. Jeaffreson says, "as much of the walls of the cyst as possible should be dissected out, or, by gently

* Medico-Chirurgical Transactions, vol. xv, p. 239; London, 1829.
† Treatise on the Diseases of the Eye, p. 701; London, 1841.
‡ Edinburgh Medical and Surgical Journal, vol. xliv, pp. 6-7; Edinburgh, 1835.

rubbing or squeezing the sides together, sufficient inflammation is induced to procure their permanent cohesion." Now, chalazion has no cyst. Molluscum, if allowed to grow very large, becomes so firmly adherent by its external surface to the neighbouring parts, that a layer of it is often left when the interior is squeezed out, and requires to be dislodged separately, which can always be effected by firm continued pressure. We have never seen encysted tumour in the eyelids, although it occurs frequently at the temporal extremity of the eyebrow. Its cyst, which Sir Astley Cooper showed to be an involution of cuticle, must be entirely removed; for if any portion of it is left, the disease will certainly be reproduced. No rubbing or squeezing the sides of it together will make them adhere.

In cases of a particle of coke fixing itself on the cornea, which is an every day railway occurrence, Mr. Jeaffreson gives the following advice:

"Great gentleness should be used in removing it; and if it is so imbedded in the cornea as not to be readily discovered or easily displaced, time must be allowed for the subsidence of the irritation, and for it to be loosened by the incipient process of ulceration." (p. 284.)

That gentleness should be used in whatever we do to the eye, we readily grant; but we have never seen any case of a particle of coke fixed on the cornea which could not be readily discovered on placing the patient in a good light, or which could not be easily displaced by using a proper instrument. A cutting instrument, such as a penknife or a lancet, should never be employed for the purpose, as it is apt to remove, with the foreign body, part of the investing membrane of the cornea, while the slightest touch with the edge of a thin spatula generally makes it start from its place. To allow any foreign body to remain on the cornea till loosened by ulceration, we should consider very bad practice; for the irritation will increase from day to day, and may end in violent inflammation.

After remarking that the removal of blood from the anterior chamber by absorption generally goes on rapidly, Mr. Jeaffreson relates the following curious example of the contrary:

"In one instance, however, in which I operated for cataract by extraction, a drop of blood was effused, and remained in the anterior chamber, which continued bright as arterial blood, and undergoing no change for several years. Sight was not entirely prevented by this drop of blood, (for it appeared as a single drop,) although slightly impeded. Year after year I observed this deposition remaining, perfectly bright, in the anterior chamber; until at last nature, as if having previously forgotten or overlooked it, seemed to take upon herself the office of removing it, and then in the course of a few weeks it rapidly disappeared." (p. 288.)

The following also appears a curious fact, whether we suppose, with our author, that the iris was actually ossified, or only that, being deprived, by some morbid process, of its colouring matter, its striated and flocculent tissue presented an appearance as if it had been converted into bone:

"In India I once had a case of ossification of the iris, in a female, of about thirty years of age, throughout its entire extent. The case was very interesting and curious: the iris was entirely converted into bone, fixed and immoveable, and the radiated structure of the bone was beautifully seen through the transparent cornea. I showed the case to many medical friends, who were much interested in its inspection; and I do not remember that any of them had seen a similar affection. The sight was but slightly impaired, nor could I trace any sufficient cause for the production of this singular phenomenon." (p. 289.)

Mr. Jeaffreson tells us that he is not aware that entozoa have been seen in the human eye by any English oculist; a confession which only shows that his studies in this branch of the subject have not been extensive enough.*

Mr. Jeaffreson describes myopia correctly; but we have never met with those individuals who, from some defect in the humours, cornea, or lens, are "possessed of a more than ordinary power of discovering distant objects." (p. 296.) Those affected with presbyopia, whom our author has here in view, do not see near objects well; but their power of discerning distant objects is just ordinary.

Mr. Jeaffreson's style is but indifferent. His language is sometimes ungrammatical, and his meaning not unfrequently obscured by faultiness of construction. He is particularly fond of separating the preposition from the noun which it governs, in order to connect different prepositions with the same word—a construction which is always inelegant. He also uses the parenthesis too often, particularly in the early part of his book, stuffing within it incidental clauses which had much better been omitted. The following sentences may serve to illustrate these defects:

"The system sympathizes more or less with the local affection, according to circumstances; chiefly to its previous state, to the degree and seat of the local affection; much, often, when the deeper-seated structures are affected; but little, generally, when the conjunctiva only is the seat of disease." (pp. 23-4.)

"The history of the case may also afford some clue; and the difference in the degree of reactive fever, greater in the former instance. (p. 85.)

"It now remains to be stated that, in a few instances, inflammation sets in with, or simultaneously affects several, not to say all, the structures of the eye." (p. 99.)

"The conjunctiva and sclerotic each exhibit various degrees of vascular congestion." (Ibid.)

"Acting in a manner somewhat similar to the effects of tumours, apoplectic seizure, or mechanical injuries, attended by the rupture of blood-vessels within the cranium, and the effusion of blood, if in such a situation as to cause pressure either on the optic nerves or the part of the brain into which they are immediately inserted, may induce amaurosis." (p. 174.)

"Previous inflammatory affections of the outer tunics of the eye, which have left nebulous or leucomatous patches on the cornea, or of the deeper-seated parts, which have gone on to the establishment of adhesions of the iris to the cornea or capsule of the lens, &c. may all militate against the success of, or positively forbid the propriety of operating, according to their degree." (p. 223.)

"I shall therefore beg leave to conclude my observations upon the diseases of the eye with a very brief notice of a few affections which have not hitherto been mentioned by me, and which may, I think, be either useful to the practitioner, or interesting to those who are more curious in the study of disease." (p. 285.)

No doubt! There are *affections which are useful to the practitioner*, but we can scarcely regard it as decorous, thus nakedly to announce the fact. It is as barefaced as if we were to say that the blunders of authors were useful to the critical profession, or that we found it to our advantage,

"With hatchets, scalping-knives in shape of pens,
To bid, like Mohocks, hapless authors die."

* Mr. Logan's case of cysticercus cellulosæ in the anterior chamber is well known. We learn that in the month of April last, a similar case occurred at Mr. Guthrie's Eye Infirmary.

